# Screening of Korean Natural Products for Anti-Adipogenesis Properties and Isolation of Kaempferol-3-*O*-rutinoside as a Potent Anti-Adipogenetic Compound from *Solidago virgaurea*

**DOI:** 10.3390/molecules21020226

**Published:** 2016-02-17

**Authors:** Young Soo Jang, Zhiqiang Wang, Jeong-Min Lee, Jae-Young Lee, Soon Sung Lim

**Affiliations:** 1 Department of Food Science and Nutrition, Hallym University, Okchon-dong, Chuncheon, Gangwon-do 200-702, Korea; ggaeby77@gmail.com (Y.S.J.); wangwq01234@gmail.com (Z.W.); 2 Department of Boichemistry, Hallym University Medical School, Okchon-dong, Chuncheon, Gangwon-do 200-702, Korea; sindelela99@nate.com (J.-M.L.); jyolee@hallym.ac.kr (J.-Y.L.); 3 Institute of Natural Medicine, Hallym University, Okchon-dong, Chuncheon, Gangwon-do 200-702, Korea

**Keywords:** obesity, screening, *Solidago virgaurea*, kaempferol-3-*O*-rutinoside

## Abstract

In this study, the anti-adipogenetic activity of 300 plant extracts was investigated using an Oil Red O staining assay in a 3T3-L1 cell line. Our results indicate that three plants, including the stem and leaf of *Physalis angulata*, the whole grass of *Solidago virgaurea*, and the root of *Dioscorea nipponica*, produced over 90% inhibition of adipogenesis. Kaempferol-3-*O*-rutinoside, which demonstrated a 48.2% inhibitory effect on adipogenesis without cytotoxicity, was isolated from the butanol layer of a water extract of *S. virgaurea* guided by the anti-adipogenesis assay in 3T3-L1. PPAR-γ and C/EBPα expression levels were determined using western blot, and our results indicate that kaempferol-3-*O*-rutinoside has a strong anti-adipogenic effect in 3T3-L1 cells through the suppression of increases in PPAR-γ and C/EBPα expression.

## 1. Introduction

Obesity is a chronic metabolic disorder caused by an imbalance between energy intake and expenditure. It is defined as abnormal or excessive fat accumulation that poses a health risk [1]. Many scientific communities have become increasingly interested in the molecular regulation of triglyceride synthesis and in phytochemical-based pharmaceutical approaches for reducing fat absorption and storage. Phytochemicals present an exciting opportunity for the discovery of new anti-obesity agents [2]. The regulation of fatty acid and triglyceride availability depends on the activity of the lipolytic enzymes of fatty acid metabolism in adipose tissue [3].

The characterization and identification of several genes involved in lipid metabolism have yielded a rich pool of potential targets for drugs to treat obesity and other metabolic syndromes [4]. One of the screening strategies used is to search for adipogenic inhibitors from plant extracts. Plants have traditionally been used as natural medicines for many diseases [5]. In particular, many oriental medicinal plants have been reported to have biological activity. Among the plant extracts screened, the extract of *Solidago virgaurea* var. *gigantea* (SV) was observed to significantly inhibit adipogenesis in 3T3-L1 adipocyte cells.

SV, a subspecies of *Solidago virgaurea* Nakai, is a perennial herb that grows on Ulreung Island in Korea. The whole plant (root and leaf) has been used as a stomachic and diuretic in Korean folk medicine, while the young aerial parts have been used as food [6]. A variety of plants belonging to the *Solidago* species has been reported to possess antibacterial, anti-oxidant [7], anti-inflammatory [8], and bone metabolic [9] activities. However, to our knowledge, no study has yet reported any anti-obesity effects of SV. In this study, we screened crude extracts from natural sources for potential anti-obesity effects on lipid accumulation in cultured 3T3-L1 adipocytes. Oil Red O staining and triglyceride contents served as indicators of lipid accumulation.

## 2. Results

### 2.1. List of Natural Extracts That Showed 30% or More Inhibition of Adipogenesis of 3T3-L1 Cells

Three-hundred crude extracts were prepared from natural plant species found in Korea or Asia, and their anti-adipogenic activity by inhibition of adipogenesis was investigated at a concentration of 10 μg/mL. The results are reported in Table 1. Among the 300 plant extracts examined, 31 crude extracts from natural plant species showed a relatively high anti-adipogenic activity (more than 30%). A significant inhibition of adipogenesis of up to 92.5% was observed with SV.

### 2.2. Effect of the S. virgaurea var. gigantea Extracts on Inhibition of 3T3-L1 Adipocyte Differentiation

**Figure 1:**
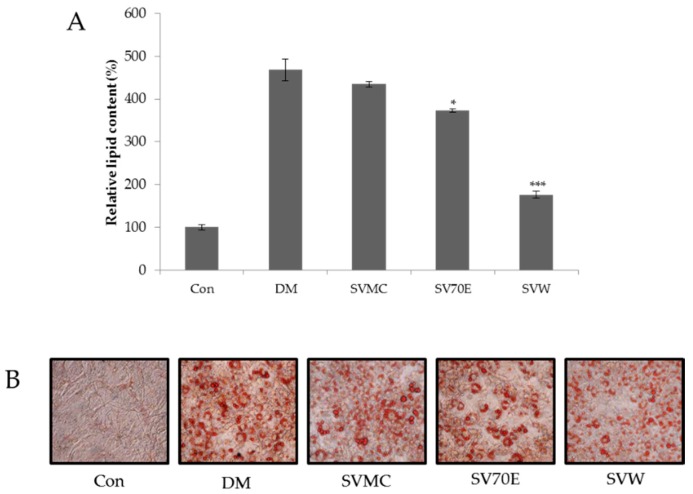
Effect of the SV extracts on inhibition 3T3-L1 adipocyte differentiation. (**A**) The relative lipid content; (**B**) Oil Red O staining images. Stained triglyceride content was quantified by measuring absorbance at 540 nm. Con is control group; DM is differentiation media cells; SVMC is methyl chloride extract of *S. virgaurea* var. *gigantea,* SV70E is 70% ethanol extract of *S. virgaurea* var. *gigantea;* SVW is water extract of *S. virgaurea* var. *gigantea.* The concentration is 10 μg/ml; three independent experiments have been carried out; * *p* < 0.05 *vs.* DM; *** *p* < 0.005 *vs.* DM.

Each extract of SV and its anti-adipogenic activity was studied at a concentration of 10 μg/mL. The results represent lipid droplet accumulation, as triglycerides in adipocytes stain with Oil Red O staining solution separate from free fatty acids and phospholipids. As shown in Figure 1, in response to the administration of SVW (water extract of SV) at 10 μg/mL, the lipid content in 3T3-L1 adipocytes decreased significantly, showing a 79.39% inhibition.

### 2.3. Effect of Solvent Fractions of SVW on Inhibition of 3T3-L1 Adipocyte Differentiation

**Figure 2:**
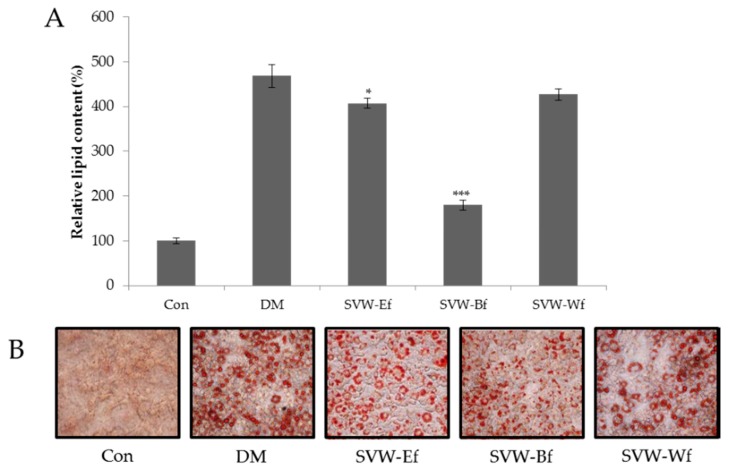
Effect of SVW solvent fractions on inhibition of 3T3-L1 adipocyte differentiation. (**A**) The relative lipid content; (**B**) Oil Red O staining images. Confluent 3T3-L1 preadipocyte were differentiated into adipocytes in medium with or without 10 μg/mL of SVW fractions for 8 day. Con is control group; DM is differentiation media cells; SVW-Ef is ethyl acetate fraction from *S. virgaurea* var. *gigantea* water extract; SVW-Bf is *n*-butanol fraction from *S. virgaurea* var. *gigante* water extract; SVW-Wf is water fraction from *S. virgaurea* var. *gigantea* water extract. Three independent experiments have been carried out; * *p* < 0.05 *vs.* DM; *** *p* < 0.005 *vs.* DM.

Solvent fractions of SVW were studied at a concentration of 10 μg/mL for inhibition of adipogenesis. As shown in Figure 2, lipid content in 3T3-L1 adipocytes decreased significantly in response to the SVW-Bf (*n*-butanol fraction of SVW) at 10 μg/mL, which showed a 78.25% inhibitory effect.

### 2.4. Effect of SVW and SVW-Bf on Preadipocyte Viability

**Figure 3:**
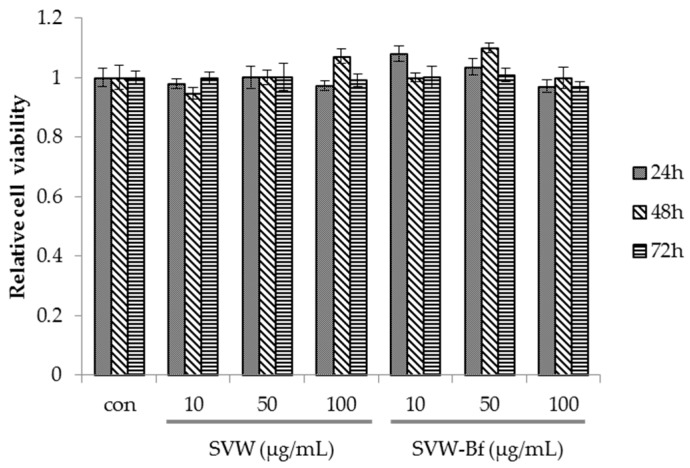
Effect of the SVW and the SVW-Bf on preadipocyte viability. 3T3-L1 preadipocytes were incubated with SVW and SVW-Bf at various concentrations (10, 50 and 100 μg/mL) for 24 h, 48 h, and 72 h. SVW is *S. virgaurea* var. *gigantea* water extracts; SVW-Bf is *n*-butanol fraction of *S. virgaurea* var. *gigante* water extract. Three independent experiments have been carried out.

An MTS assay was performed to assess the effect of the SVW and SVW-Bf on 3T3-L1 cell viability. As shown in Figure 3, the SVW and SVW-Bf at 10, 50, and 100 μg/mL had no significant effect on viability after 72 h of treatment. The cells did not become toxic, even when the SVW and SVW-Bf were added at the highest concentration (100 μg/mL) for 72 h.

### 2.5. Effect of Sub-Fractions of SVW-Bf on Inhibition of 3T3-L1 Adipocyte Differentiation

**Figure 4:**
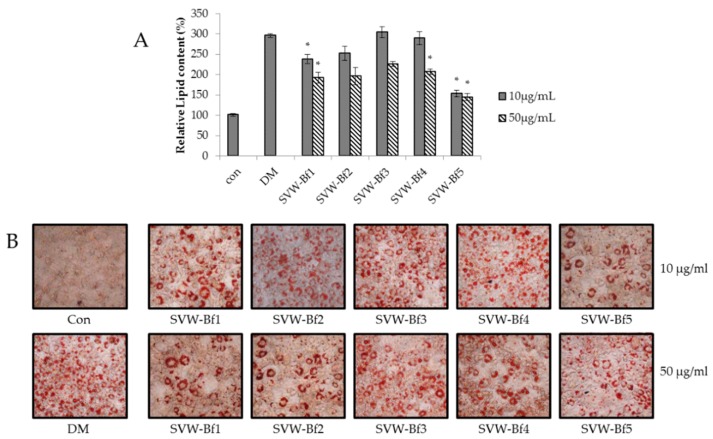
Effect of Diaion HP-20 fractions of SVW-Bf in 3T3-L1. (**A**) The relative lipid content; (**B**) Oil Red O staining images. Confluent 3T3-L1 preadipocytes were differentiated into adipocytes in medium with or without different concentrations of SVW-Bf Diaion HP-20 fractions for 8 days. Three independent experiments have been carried out; * *p* < 0.05 *vs.* DM.

The SVW-Bf produced a significant anti-adipogenic effect at 10 μg/mL. Therefore, for further study, we fractionated the SVW-Bf on an open column using Diaion HP-20 resin. We obtained five fractions from the SVW-Bf, and their adipogenesis inhibitory effects were assessed. As shown in Figure 4, Diaion HP-20 fraction 5 of SVW-Bf (SVW-Bf5) produced a significant anti-adipogenic effect at 10 μg/mL (72.78%) and 50 μg/mL (77.17%).

### 2.6. Effect of Kaempferol-3-O-rutinoside, Chlorogenic Acid and Protocatechuic Acid of SVW-Bf5 on Preadipocyte Viability and Inhibition of 3T3-L1 Adipocyte Differentiation

**Figure 5:**
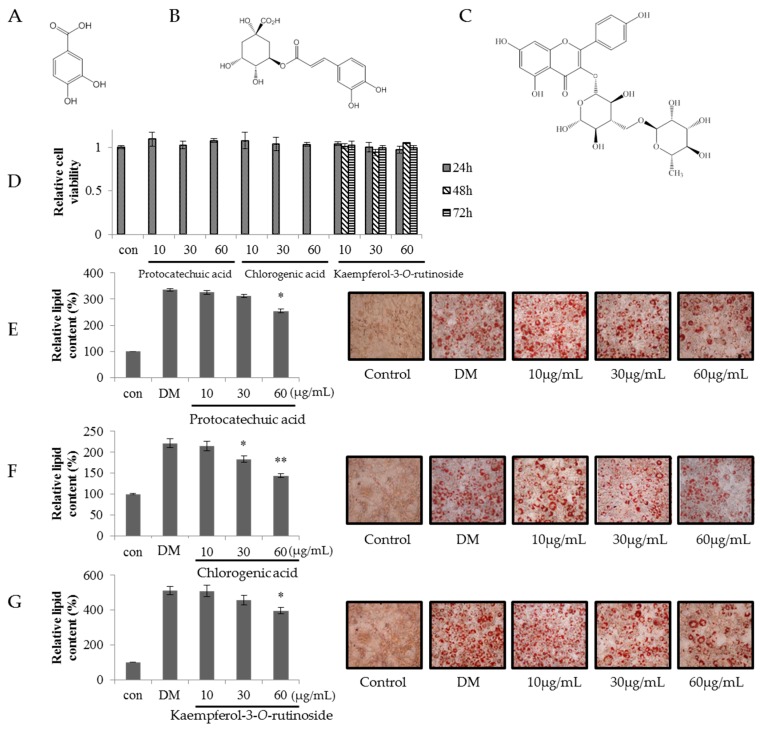
Molecular structures of (**A**) protocatechuic acid; (**B**) chlorogenic acid; and (**C**) kaempferol-3-*O*-rutinoside; (**D**) preadipocyte viability effect of protocatechuic acid, chlorogenic acid and kaempferol-3-*O*-rutinoside; relative lipid content and Oil Red O staining images of (**E**) protocatechuic acid; (**F**) chlorogenic acid; and (**G**) kaempferol-3-*O*-rutinoside. Three independent experiments have been carried out; * *p* < 0.05 *vs.* DM; ** *p* < 0.01 *vs.* DM.

Kaempferol-3-*O*-rutinoside (**K-3-*O*-R**), chlorogenic acid (**CA**) and protocatechuic acid (**PA**), which were major components isolated from SVW-Bf5, were studied for inhibition of adipogenesis at a concentration of 10, 30 and 60 μg/mL. As shown in Figure 5, lipid content in 3T3-L1 adipocytes decreased significantly in response to the **K-3-*O*-R** (48.2%), **CA** (63.9%) and **PA** (66.8%) at 60 μg/mL.

### 2.7. Effect of Kaempferol-3-O-rutinoside on PPAR-γ and C/EBP-α Protein Expression in 3T3-L1 Cells

**Figure 6:**
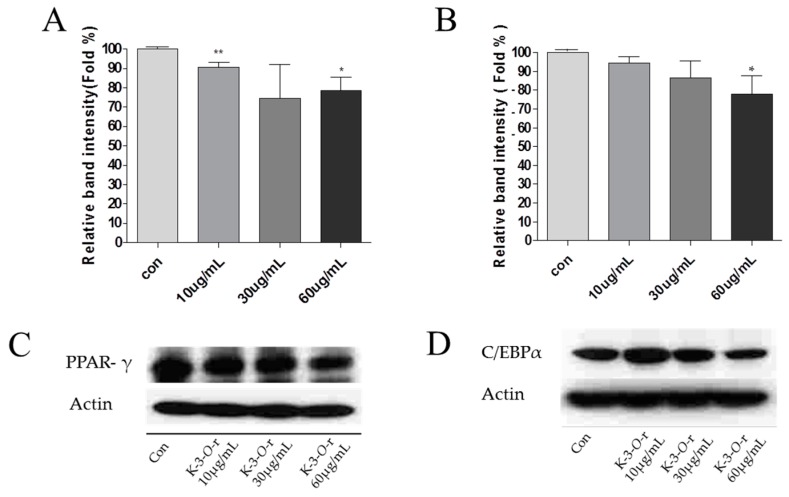
Effect of kaempferol-3-*O*-rutinoside on PPAR-γ (**A**,**C**) and C/EBP-α (**B**,**D**) expression in 3T3-L1 cells. Three independent experiments have been carried out; * *p* < 0.05 *vs.* DM; ** *p* < 0.01 *vs.* DM. mL not ml.

**K-3-*O*-R**, a major component of SV extract, decreased the expression levels of known adipogenesis markers, peroxisome proliferative-activated receptor γ (PPAR-γ) and CCAAT/ enhancer-binding protein α (C/EBPα), during the adipogenesis of 3T3-L1 cells in a concentration-dependent manner (Figure 6). We demonstrated a decrease in PPAR-γ and C/EBPα by **K-3-*O*-R** in 3T3-L1 cells. PPAR-γ [10] and C/EBPα [11] increased in 3T3-L1 cells as part of transcriptional regulation of differentiation. However, PPAR-γ and C/EBPα expression decreased after **K-3-*O*-R** treatment in 3T3-L1 cells. Therefore, treatment of **K-3-*O*-R** suppresses adipogenesis and 3T3-L1 cell differentiation.

## 3. Discussion

Obesity is quickly becoming one of the most serious health problems leading to death in modern society, owing to the consequent increased risk of hypertension, hyperlipidemia, cardiovascular diseases, diabetes, cancers, and non-alcoholic fatty liver disease [12]. It is generally associated with an excessive growth of adipose tissue mass caused by an increase in the number and size of fat cells, so the amount of adipose tissue mass can be regulated by the suppression of adipogenesis [13,14,15]. Thus, obesity could be prevented by lose weight through diet, physical activities, treatment with anti-adipogenesis agents, *etc.* [16,17]. To develop anti-adipogenesis agents, 3T3-L1 cells have served as a well-documented model system [18]. A promising source of such agents appears to come from Nature [1].

Using the 3T3-L1 model of adipogenesis, the anti-obesity activity of 300 crude extracts was screened at a concentration level of 10 μg/mL. This revealed 33 of 300 extracts that were active at 30% inhibition and greater. Among them, nine belonged to the Compositae family, five to the Liliaceae family, four to the Convolvulaceae family, three to the Eucommiaceae family, and three to the Solanaceae family. Their activities are hypothesized to be family-dependent due to their similar metabolic compositions. Out of these 31 plants, three, including the stem and leaf of *Physalis angulata*, the whole grass of *Solidago virgaurea*, and the root of *Dioscorea nipponica*, presented strong (over 90%) inhibition of adipogenesis in 3T3-L1 cells. The anti-obesity effect of *Physalis angulata* and *Dioscorea nipponica* have been reported in previous studies [19,20]. However, there have been no previous investigations of SV and its anti-adipogenesis activity. Thus, this plant could represent a new potential source of anti-adipogenesis activity and was selected for further study, including active component screening.

SV (European goldenrod or woundwort) is an herbaceous perennial plant that has been traditionally used to treat urinary tract, nephrolithiasis, and prostate pathologies [21]. Its antioxidant activities and anti-cardiotoxicity effects have also been previously reported [22]. In the present study, we attempted to identify and isolate the active components of SV guided by an anti-adipogenic assay in 3T3-L1 cells, and **K-3-*O*-R**, which demonstrated a 48.2% inhibitory effects at 60 μg/mL without cytotoxicity within 72 h in accordance with previous results [23], was obtained from the SVW-Bf5. Interestingly, the inhibitory activity of **K-3-*O*-R** is no more than those of crude extract and fractions. Hence, synergistic effects and/or other efficient anti-adipogenesis components would be presented. In order to recognize whether synergistic effect it is, another two major components were isolated from SVW-Bf5, which are CA and PA also showed lower inhibitory than crude extracts. This seems to suggest that these results would be the result of a synergistic effect, which is a widespread phenomenon in natural products [24]. Scazzocchio *et al.* have reported a similar effect in the same pathway [25]. 

**K-3-*O*-R** is known as potent α-glucosidase inhibitor that exists in many plants [26]. As a flavonol glucoside, **K-3-*O*-R** would be ingested as a glycoside and adsorbed in the systemic circulation as **K-3-*O*-R**, kaempferol, kaempferol conjugated forms and other phenolic acids, and then arrive at target tissues [27]. A previous study has demonstrated that kaempferol inhibits lipid accumulation in adipocytes and zebrafish, and attenuates the late adipogenic factors such as PPAR-γ and C/EBPα [28]. In order to understand the mechanism of anti-adipogenesis action of **K-3-*O*-R** in 3T3-L1 cells, its effect on PPAR-γ and C/EBPα was studied in the present work. The biochemical pathways of adipogenesis in the 3T3-L1 cell line have been well characterized [29]. The transcription factors PPAR-γ and C/EBPα play key roles in the complex transcriptional cascade of adipocyte differentiation that will eventually activate and express adipocyte-specific genes such as fatty acid synthetase, fatty acid binding protein, leptin, adiponectin and *etc.*, which closely related to obesity disease such as diabetes and non-alcoholic fatty liver disease [30]. PPAR-γ [10] and C/EBPα [11] are known to increase in 3T3-L1 cells as part of transcriptional regulation of differentiation; however, their levels were decreased after treatment with **K-3-*O*-R** in 3T3-L1 cells. Our results indicate that **K-3-*O*-R** isolated from SV has a strong anti-adipogenic effect in 3T3-L1 cells by suppressing the expression of PPAR-γ and C/EBPα.

## 4. Materials and Methods

### 4.1. General Information

Diaion HP-20 resin and sephadex LH-20 used for separation were purchased from Sigma-Aldrich (St. Louis, MO, USA) and GE Healthcare (Uppsala, Sweden), respectively. All organic solvents used for extraction and isolation were obtained from Samchun (Pyeongtaek-si, Gyeonggi-do, Korea). Ultrapure water used for extraction, isolation and all solutions was obtained using a Milli-Q laboratory water pufification system (Millipore, Bedford, MA, USA) with a resistivity over 18.2 MΩ·cm. Fourier-transform-NMR spectrometer used for nuclear magnetic resonance were obtained from Bruker Korea (Seongnam, Korea). Signal processing and interpretation were performed using the Bruker DPX 400 MHz (9.4T) package. JMS-700 Mstation used for EI-MS were obtained from JEOL (Tokyo, Japan). Voyager-DE-STR-MALDI-TOF Mass Spectrometer used for MALDI-TOF MS was purchased from Applied Biosysterms (Foster City, CA, USA).

### 4.2. Plant Materials

All 300 medicinal plants used for anti-adipogenesis screening were selected from the sample bank of the Laboratory of Natural Products Chemistry, Department of Food Science and Nutrition, Hallym University, Chuncheon, Republic of Korea. SV used for further screening and isolation was supplied by the Agriculture Technology Center of Ulreung Island, Korea. A voucher sample (RIC-2000-10) was deposited at the Center for Efficacy Assessment and Development of Functional Foods and Drugs, Hallym University. The specimens were authenticated by Emeritus Professor H.J. Chi, Seoul National University, Seoul, Korea.

### 4.3. Preparation of Natural Extracts

The natural products were extracted in water or ethanol, by using evaporative solvent removal. The concentrated samples were stored at –20 °C for further study.

### 4.4. Preparation of S. virgaurea var. gigantea

**Figure 7:**
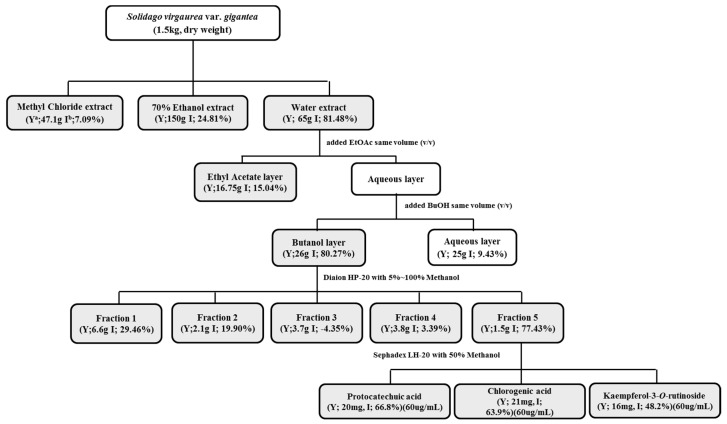
Extraction and fractionation of SV. ^a^ Yield; ^b^ Inhibition % (concentrations of protocatechuic acid, chlorogenic acid, kaempferol-3-*O*-rutinoside are 50 μg/mL; concentrations of others are 10 μg/mL).

SV (1.5 kg) was extracted twice with chloroform (15 L, room temperature) at for 48 h. After filtration, the dried SV was extracted with 70% ethanol (EtOH, 15 L, room temperature) twice for 48 h. Afterwards, the dried residue was extracted two times with water (15 L) for 2 h at 100 °C. The active water extract (SVW) was suspended in distilled water and partitioned with ethyl acetate (EtOAc) and *n*-butanol (*n*-BuOH) to yield an EtOAc fraction (SVW-Ef, 16.75 g), an *n*-BuOH fraction (SVW-Bf, 26 g), and a water fraction (SVW-Wf, 25 g). The active SVW-Bf was subfractionated using Diaion HP-20 resin with 20%, 40%, 60%, 80%, and 100% methanol, and five fractions were obtained: fraction 1 (SVW-Bf1, 6.6 g), fraction 2 (SVW-Bf2, 2.1 g), fraction 3 (SVW-Bf3, 3.7 g), fraction 4 (SVW-Bf4, 3.8 g), and fraction 5 (SVW-Bf5, 1.5 g). Finally, K-3-*O*-R, CA, and PA were isolated as active principle compounds from SVW-Bf5 by Sephadex LH-20 with 50% MeOH guided by Oil Red O staining in 3T3-L1 cells (Figure 7).

The structures of the isolated compounds were identified by mass spectrometry and ^1^H-NMR as follows:

*Kaempferol-3-O-rutinoside* (**K-3-*O*-R**); EI-MS *m*/*z*: 287 [M − H]^−^; ^1^H-NMR (400 MHz CD_3_OD): δ 6.21 ppm (br s, H-6), 6.40 ppm (br s, H-8), 8.06 ppm (d, *J* = 9.0 Hz, H-2′/H-6′), 6.90 ppm (d, *J* = 9.0 Hz, H-3′/H5′), 5.11 ppm (d, *J* = 7.5 Hz, H-1′′), 4.52 ppm (br s, H-1′′′), 1.12 ppm (d, *J* = 6.0 Hz, H-6′′′), 3.27–3.80 ppm (H-2′′ to H-6′′, H-2′′′to H-5′′′). The MS and ^1^H-NMR data for K-3-*O*-R are identical to those reported previously [31]. 

*Chlorogenic acid* (**CA**); MALDI-TOF MS *m*/*z*: 377 [M + Na]^+^; ^1^H-NMR (400 MHz CD_3_OD): δ 7.55 ppm (1H, d, *J* = 15.9 Hz, H-7′), 7.04 ppm (1H, d, *J* = 1.8 Hz, H-2′), 6.94 ppm (1H, dd, *J* = 8.2 Hz and *J* = 1.8 Hz, H-6′), 6.77 ppm (1H, d, *J* = 8.2 Hz, H-5), 6.26 ppm (1H, d, *J* = 15.9 Hz, H-8′), 3.72 ppm (1H, m, H-3), 2.21 ppm (2H, m, H-6), 2.05 ppm (2H, m, H-2). The MS and ^1^H-NMR data for CA are identical to those reported previously [32,33].

*Protocatechuic acid* (**PA**); EI-MS *m*/*z*: 154 [M]^+^; ^1^H-NMR (400 MHz CD_3_OD): δ 7.43 ppm (1H, d, *J* = 2.0 Hz, H-2), 7.42 ppm (1H, dd, *J* = 8.0 and 2.0 Hz, H-6), and 6.79 ppm (1H, d, *J* = 8.2 Hz, H-5). The MS and ^1^H-NMR data for PA are identical to those reported previously [34].

### 4.5. Cell Culture and Differentiation

3T3-L1 fibroblasts were obtained from ATCC (Manassas, VA, USA) and grown at 37 °C under a humidified 5% CO_2_ atmosphere in Dulbecco’s modified Eagle’s medium (DEME, Gibco, Waltham, MA, USA) containing 10% bovine calf serum (GenDEPOT, Katy, TX, USA) and 100 U/mL penicillin-streptomycin (Gibco). Two days after confluence, preadipocytes of 3T3-L1 (designated as day 0) were cultured in differentiation medium (DM) containing 10% fetal bovine serum (FBS, Gibco), 10 μg/mL insulin (Sigma-Aldrich), 0.5 mM isobutylmethyxanthine (Sigma-Aldrich), and 1 µM dexamethasone (Sigma-Aldrich). After 2 days, the medium was switched to post-DM containing 10% FBS and 10 μg/mL insulin for 4 days, and then changed to 10% FBS medium for an additional 2 days to induce differentiation.

### 4.6. Oil Red O Staining

SV extracts and its solvent fractions treated to 3T3-L1 cells at the concentration of 10 μg/mL on the day 4 after differentiation induction; Diaion HP-20 fractions of SVW-Bf treated to 3T3-L1 cells at the concentration of 10 and 50 μg/mL; each isolated compound treated to 3T3-L1 cells at the concentration of 10, 30, and 60 μg/mL. The Oil Red O staining was performed on the day 8 after differentiation induction as following. Briefly, the 3T3-L1 adipocyte cells were washed with phophate buffered saline (PBS) and fixed with 10% formalin. After the Oil Red O staining, cells were photographed using a phase-contrast microscope (Olympus CKX41, Tokyo, Japan) in combination with a digital camera (Canon Inc., Tokyo, Japan) at 200× magnification. The lipid droplets were dissolved in isoprapanol and measure at 540 nm using a microplate reader (Sensident scan, Labsystems, Helsinki, Finland). The relative lipid content and adipogensis inhibitory percentage were calculated using the following equations:

Relative lipid content (%) = (Sample O.D/Control O.D) × 100%(1)

Inhibition (%) = {1 − (Sample O.D − Control O.D)/(DM O.D − Control O.D)} × 100%)(2)

### 4.7. Cell Viability Assay

The cell viability was performed using an MTS [3-(4,5-dimethylthiazol-2-yl)-5-(3-carboxy-methoxyphenyl)-2-(4-sulfophenyl)-2*H*-tetrazolium, inner salt] assay kit (Promega, Madison, WI, USA). Briefly, 3T3-L1 cells were seeded into 96-well plates (5 × 10^3^ cells/well) and treated with various concentrations of SVW (10, 50, and 100 μg/mL), SVW-Bf (10, 50, and 100 μg/mL), and kaempferol-3-*O*-rutinoside (10, 30, and 60 μg/mL) for 24 h, 48 h, and 72 h, respectively; treated with chlorogenic acid (10, 30, and 60 μg/mL) and protocatechuic acid (10, 30, and 60 μg/mL) for 24 h. After incubation, 20 μL/well of MTS solution and incubated for 20 min at 37 °C in a humidified 5% CO_2_ atmosphere. The optical density at 490 nm was measured three times using a microplate reader (Sensident scan).

### 4.8. Western Blot Analysis

3T3-L1 cells pads were prepared using lysis buffer (10 mM Tris–HCl, pH 7.4, 100 mM NaCl, 5 mM EDTA, 10% glycerol, and 1% NP-40, 0.1 mM PMSF, 10 μg/mL each of leupeptin, aprotinin, and pepstatin A). Protein (25 μg) was separated by 8% SDS-polyacrylamide gel electrophoresis, transferred to polyvinylidene difluoride membranes (Millipore, Billerica, MA, USA), and hybridized overnight with 1:1000 diluted PPAR-γ C/EBPα and Actin primary antibodies (PPAR-γ; Cell Signaling Technology, Danvers, MA, USA, C/EBPα; Cell Signaling Technology, Actin; Sigma-Aldrich). After incubation with 1:2000 diluted horseradish-peroxidase conjugated goat anti-mouse or donkey anti-rabbit secondary antibody (Cell Signaling Technology) for 1 h at room temperature, the immunoreactive protein were visualized by ECL system (Amersham Biosciences, Pittsburgh, PA, USA) and quantified using a densitometric analysis.

### 4.9. Statistical Analysis

All values are mean ± S.E.M. For statistical analysis, the *p* value was calculated using a two-tailed unpaired Student’s *t*-test with *p* < 0.05 considered statistically significant.

## Figures and Tables

**Table 1:** List of natural extracts that demonstrate 30% or more inhibition of adipogenesis in 3T3-L1 cells. 3T3-L1 cells differentiated with differentiation media in the absence or presence of natural extracts for 8 days (concentration: 10 μg/mL).

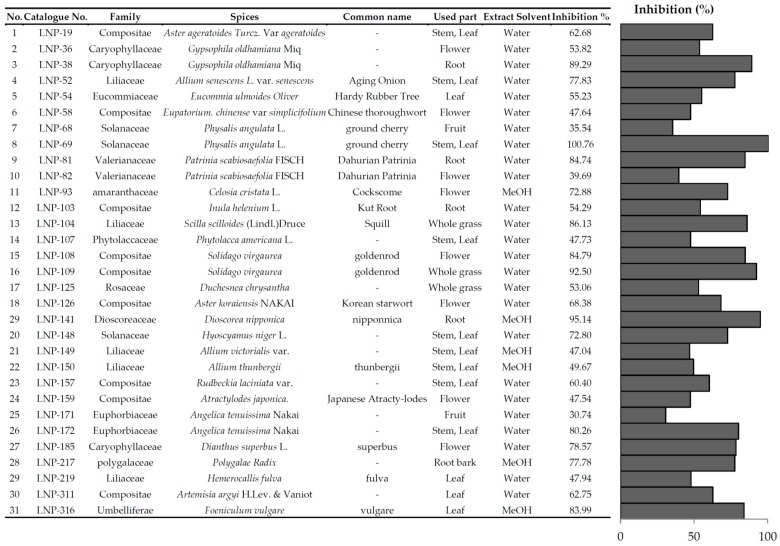

## References

[1] Kopelman P.G. (2000). Obesity as a medical problem. Nature.

[2] Bray G.A., Tartaglia L.A. (2000). Medicinal strategies in the treatment of obesity. Nature.

[3] Vázquez-Vela M.E. F., Torres N., Tovar A.R. (2008). White adipose tissue as endocrine organ and its role in obesity. Arch. Med. Res..

[4] Reilly S.M., Lee C.-H. (2008). PPARδ as a therapeutic target in metabolic disease. FEBS Lett..

[5] Li S., Luo X. (2003). Compendium of Materia Medica: (Bencao Gangmu).

[6] Lee T.B. (1979). Illustrated Flora of Korea.

[7] Kim H.S. (1996). Studies on the antimicrobial and antioxidant activity of *Solidago virga-aurea* LI and *Solidago virgaurea* Linne var. *asiatica* Nakai. MS Thesis.

[8] Leuschner J. (1995). Anti-inflammatory, spasmolytic and diuretic effects of a commercially available *Solidago gigantea* Herb. extract. Arzneimittelforschung.

[9] Lee J. (2004). Effect of *Solidago Virga-aurea* var. *giagantea* Mig. Root extract on the activity of osteoblastic cells and bone metabolism. MS Thesis.

[10] Watanabe M., Inukai K., Katagiri H., Awata T., Oka Y., Katayama S. (2003). Regulation of PPARγ transcriptional activity in 3T3-L1 adipocytes. Biochem. Biophys. Res. Commun..

[11] Cao Z., Umek R.M., McKnight S.L. (1991). Regulated expression of three C/EBP isoforms during adipose conversion of 3T3-L1 cells. Genes Dev..

[12] Tarantino G. (2007). Should nonalcoholic fatty liver disease be regarded as a hepatic illness only?. World J. Gastroenterol..

[13] Yang J.Y., Della-Fera M.A., Rayalam S., Ambati S., Hartzell D.L., Park H.J., Baile C.A. (2008). Enhanced inhibition of adipogenesis and induction of apoptosis in 3T3-L1 adipocytes with combinations of resveratrol and quercetin. Life Sci..

[14] Rosen E.D., Spiegelman B.M. (2006). Adipocytes as regulators of energy balance and glucose homeostasis. Nature.

[15] Roncari D.A.K., Lau D.C.W., Kindler S. (1981). Exaggerated replication in culture of adipocyte precursors from massively obese persons. Metabolism.

[16] Finelli C., Tarantino G. (2012). Is there any consensus as to what diet or lifestyle approach is the right one for NAFLD patients?. J. Gastrointestin. Liver Dis..

[17] Seo J.B., Choe S.S., Jeong H.W., Park S.W., Shin H.J., Choi S.M., Park J.Y., Choi E.W., Kim J.B., Seen D.S. (2011). Anti-obesity effects of *Lysimachia feonum-graecum* characterized by decreased adipogenesis and regulated lipid metabolism. Exp. Mol. Med..

[18] Kong C.S., Kim J.A., Kim S.K. (2009). Anti-obesity effect of sulfated glucosamine by AMPK signal pathway in 3T3-L1 adipocytes. Food Chem. Toxicol..

[19] Kwon C.-S., Sohn H.Y., Kim S.H., Kim J.H., Son K.H., Lee J.S., Lim J.K., Kim J.-S. (2003). Anti-obesity effect of *Dioscorea nipponica* Makino with lipase-inhibitory activity in rodents. Biosci. Biotechnol. Biochem..

[20] Lahrita L., Kato E., Kawabata J. (2015). Uncovering potential of Indonesian medicinal plants on glucose uptake enhancement and lipid suppression in 3T3-L1 adipocytes. J. Ethnopharmacol..

[21] Demir H., Açik L., Bali E.B., Koç L.Y., Kaynak G. (2009). Antioxidant and antimicrobial activities of *Solidago virgaurea* extracts. Afr. J. Biotechnol..

[22] El-Tantawy W.H. (2014). Biochemical effects of *Solidago virgaurea* extract on experimental cardiotoxicity. J. Physiol. Biochem..

[23] Jafari S., Saeidnia S., Hajimehdipoor H., Ardekani M.R.S., Faramarzi M.A., Hadjiakhoondi A., Khanavi M. (2013). Cytotoxic evaluation of *Melia azedarach* in comparison with, *Azadirachta indica* and its phytochemical investigation. DARU.

[24] Christensen K.B., Petersen R.K., Kristiansen K., Christensen L.P. (2010). Identification of bioactive compounds from flowers of black elder (*Sambucus nigra* L.) that active the human peroxisome proliferator-active receptor (PPAR) γ. Phytother. Res..

[25] Scazzocchio B., Varì R., Filesi C., D’Archivio M., Santangelo C., Giovannini C., Iacovelli A., Silecchia G., Volti G.L., Galvano F. (2011). Cyanidin-3-*O*-β-glucoside and protocatechuic acid exert insulin-like effects by upregulating PPARγ activity in human omental adipocytes. Diabetes.

[26] Habtemariam S. (2011). α-Glucosidase inhibitory activity of kaempferol-3-*O*-rutinoside. Nat. Prod. Commun..

[27] Calderón-Montaño J.M., Burgos-Morón E., Pérez-Guerrero C., López-Lázaro M. (2011). A review on the dietary flavonoid kaempferol. Mini-Rev. Med. Chem..

[28] Lee Y.J., Choi H.S., Seo M.J., Jeon H.J., Kim K.J., Lee B.Y. (2015). Kaempferol suppresses lipid accumulation by inhibiting early adipogenesis in 3T3-L1 cells and zebrafish. Food Funct..

[29] Gregoire F.M., Smas C.M., Sul H.S. (1998). Understanding adipocyte differentiation. Physiol. Rev..

[30] Finelli C., Tarantino G. (2013). What is the role of adiponectin in obesity related non-alcoholic fatty liver disease?. World J. Gastroenterol..

[31] Takemura T., Takatsu Y., Kasumi M., Marubashi W., Iwashina T. (2005). Flavonoids and their distribution patterns in the flowers of *Gladiolus* cultivars. Acta Hortic..

[32] Leiss K.A., Maltese F., Choi Y.H., Verpoorte R., Klinkhamer P.G.L. (2009). Identification of chlorogeneic acid as a resistance factor for thrips in chrysanthemum. Plant Physiol..

[33] Jung H.A., Park J.C., Chung H.Y., Kim J., Choi J.S. (1999). Antioxidant flavonoids and chlorogenic acid from the leaves of *Eriobotrya japonica*. Arch. Pham. Res..

[34] Lee J.C., Lee K.Y., Na C.S., Jung N.C., Chung G.H., Jang Y.S. (2004). Extract from *Rhus verniciflua* Stokes is capable of inhibiting the growth of human lymphoma cells. Food Chem. Toxicol..

